# Effect of freezing and thawing on ejaculated sperm and subsequent pregnancy and neonatal outcomes in IVF

**DOI:** 10.3389/fendo.2024.1408662

**Published:** 2024-12-16

**Authors:** Qin Xie, Xueyi Jiang, Ming Zhao, Yating Xie, Yong Fan, Lun Suo, Yanping Kuang

**Affiliations:** ^1^ Department of Assisted Reproduction, Shanghai Ninth People’s Hospital, Shanghai Jiao Tong University School of Medicine, Shanghai, China; ^2^ Department of Assisted Reproduction, Shanghai Towako Hospital, Shanghai, China; ^3^ Department of Reproductive Medicine, Kunming Angel Women & Children’s Hospital, Kunming, Yunnan, China

**Keywords:** frozen sperm, *in vitro* fertilization, assisted reproduction, pregnancy outcomes, neonatal outcomes

## Abstract

**Background:**

Techniques for sperm cryopreservation have exhibited their potential in male fertility preservation. The use of frozen–thawed sperm in *in vitro* fertilization (IVF) cycles is widespread today. However, many studies reported that cryopreservation might have adverse effects on sperm DNA integrity, motility, and fertilization, probably due to cold shock, intra- and extracellular ice crystals, and excess reactive oxygen species (ROS). Studies suggested that freezing and thawing impaired sperm viability and might adversely affect subsequent fertilization and pregnancy outcomes. The potential damage to fertilization and subsequent embryonic development and offspring health raises the concern on sperm cryopreservation. However, the above mentioned studies are limited to intracytoplasmic sperm injection (ICSI) cycles, while IVF is a more natural and patient-friendly method. IVF requires a higher quality of sperm than ICSI. However, the effect of freezing and thawing on sperm used for IVF remains unknown. Therefore, we aim to investigate the effect of freezing and thawing on ejaculated sperm and subsequent pregnancy and neonatal outcomes in IVF.

**Methods:**

This retrospective cohort study at a tertiary-care academic medical center included 447 women who used paternal frozen–thawed ejaculated sperm and 31,039 women who used paternal freshly ejaculated sperm for IVF and underwent frozen–thawed blastocyst transfer from January 2011 to September 2021. To balance the baseline characteristics of the two groups, patients using frozen sperm were matched with control groups using a propensity score matching algorithm with a ratio of 1:3.

**Results:**

Although sperm motility decreased from 82.04% to 75.70% (P < 0.001) after the freezing–thawing process, the fertilization rate (68.27% for frozen sperm and 67.54% for fresh sperm), number of viable embryos (1.98 and 2.16), clinical pregnancy rate (44.7% and 51.8%), and live birth rate (40.3% and 42.4%) were comparable between the two groups (all P > 0.05). For neonatal outcomes, no between-group differences were observed in offspring gender, gestational age, birthweight, and the rate of preterm birth (21.7% and 12.9%), low birthweight neonates (19.2% and 16.0%), and birth defects (0.0% and 0.8%) (all P>0.05).

**Conclusions:**

Frozen–thawed sperm had lower sperm motility but resulted in comparable embryonic, pregnancy, and neonatal outcomes versus fresh sperm in IVF cycles.

## 1 Introduction

Since the first successful pregnancy from frozen–thawed human sperm (1), cryopreservation of sperm has helped thousands of patients with azoospermia and severe oligozoospermia (2, 3). Thanks to the rapid development of cryopreservation techniques, the applied range of frozen sperm has expanded to a larger population nowadays and made it possible for male fertility conservation, sperm donation, and regular assisted reproductive technology (ART) treatment (4, 5).

Application of frozen–thawed sperm in regular *in vitro* fertilization (IVF) treatments is considered essential and patient-friendly. Freezing sperm in advance can offer both patients and doctors flexible schedules, avoid oocyte waste in case of unpredictable early ovulation, and help relieve tensions that may lead to failed sperm retrieval.

However, some studies have pointed out that the freezing–thawing process would impair sperm motility, DNA integrity, and fertilization capacity, probably due to cold shock, intracellular and extracellular ice crystals, and excessive ROS (6–14). Meanwhile, some clinical studies focused on intracytoplasmic sperm injection (ICSI) cycles suggested that the freezing–thawing process did impair sperm motility but had no detrimental effect on subsequent fertilization and pregnancy outcomes (15–18). Compared with ICSI, IVF is a more natural and patient-friendly way but has a higher requirement for sperm density and motility. Some studies showed a little lower live birth rate in IVF cycles than in ICSI cycles (11–13) (19). However, to our best knowledge, the effect of the freezing–thawing process on clinical outcomes in IVF cycles remained unclear.

Taken together, the aim of this study is to assess the effect of frozen–thawed sperm in IVF cycles on embryonic, pregnancy, and neonatal outcomes.

## 2 Materials and methods

### 2.1 Ethical approval

This study was approved by the Ethics Committee (Institutional Review Board) of the hospital (SH9H-2020-T400-1).

### 2.2 Study design and patients

We performed this retrospective cohort study at the Department of Assisted Reproduction of Shanghai Ninth People’s Hospital affiliated with Shanghai Jiao Tong University School of Medicine. We studied all women who underwent IVF with paternal ejaculated sperm and subsequent frozen–thawed blastocyst transfer from January 2011 to September 2021. After excluding patients with core data missing or repeated cycles during the study period, women using frozen–thawed sperm were matched with those who used fresh sperm through propensity score matching.

### 2.3 Sperm cryopreservation and laboratory protocols

The standard operating procedure of freezing and thawing sperm in our center remained unchanged during the study period. To freeze sperm, we added an equal volume of cryoprotectant to ejaculated sperm and placed it at 4°C for 20 min at first and then in liquid nitrogen vapor for 10 min. The sperm was immersed in liquid nitrogen until use. To thaw sperm, the samples were placed at 37°C for 3 min. For semen preparation, fresh sperm was prepared after liquefaction, and frozen sperm was prepared after thawing. First, semen was centrifuged using isolate UP and isolate DOWN. Then, we added 1-mL human tubal fluid (HTF; Irvine Scientific, USA) with 5%(v/v) serum substitute supplement (SSS; Irvine Scientific, USA) to the pellet and let the sperm swim up at 37° for 60 min. The sperm quality was assessed under a microscope.

IVF was performed 4–6 h after oocyte retrieval. Sperm was added into the drop of HTF with 10% (v/v) SSS. Fertilization was assessed 16–20 h later, and two pronucleus would be considered as normal fertilization. The zygotes were cultured in a humidified atmosphere containing 5% O_2_ and 6% CO_2_ at 37°C.

The endometrial preparation for FET has been previously reported in detail (20). Briefly, women with regular ovulatory cycles, irregular menses, or a history of thin endometrium underwent modified natural cycles, mildly stimulated cycles, or artificial cycles, respectively.

As previously reported (21), embryo vitrification was performed with a Cryotop carrier system (Kitazato Biopharma Co.), and dimethyl-sulfoxide-ethylene glycol-sucrose was used as cryoprotectants. A descending concentration gradient of sucrose (1 to 0.5 to 0 mol/L) was used for embryo thawing.

### 2.4 Outcome measures and definitions

The main outcomes included fertilization rate (%), number of viable embryos, clinical pregnancy rate (%), and live birth rate (%). Other outcomes included concentration and motility after freezing-thawing process, viable rate per oocyte retrieved (%), number of good embryos, good embryos rate per oocyte retrieved (%), biochemical pregnancy rate (%), implantation rate (%), and miscarriage rate (%).

Normal fertilization was defined as the presence of two pronucleus after IVF. Clinical pregnancy was defined as the presence of a gestational sac as detected by ultrasound examination at 7 weeks post–FET. The implantation rate was defined as the proportion of the number of gestational sacs among the number of embryos transferred. Live birth was defined as the delivery of any viable infant at more than 28 weeks of gestation.

### 2.5 Statistical analysis

We performed propensity score matching to account for differences in baseline characteristics. Women using frozen sperm were matched with controls who used fresh sperm by using the nearest matching pattern with a ratio of 3. The propensity scores were calculated by logistic regression with 10 covariates including female age (continuous), male age (continuous), female BMI (continuous), type of infertility (primary or secondary), infertility duration (continuous), gravidity (0 or ≥1), parity (0 or ≥1), female infertility diagnosis (tubal, mixed/other factors, unexplained), sperm concentration before preparation (continuous), sperm motility before preparation (continuous), treatment protocol (PPOS, mild stimulation, GnRH-ant protocol, long protocol, short protocol and natural cycles), treatment year (2011–2013, 2014–2015, 2016–2018 and 2019–2020), and number of oocytes retrieved (continuous).

Continuous variables were presented as mean with standard deviation, and categorical variables were presented as frequencies with percentages. First, we test normality by the Shapiro–Wilk test and the homogeneity of variances by Levene’s test. According to the results of the above mentioned tests, continuous variables were compared by using *t*-test or *U*-test as appropriate. For categorical variables, between-group differences were analyzed by using the chi-square test or Fisher exact test as appropriate.

Statistical analysis was performed using R statistical programming language (version 4.0.3; R Foundation for Statistical Computing, Vienna, Austria), and statistical significance was set at *P* < 0.05.

## 3 Results

**Figure 1:**
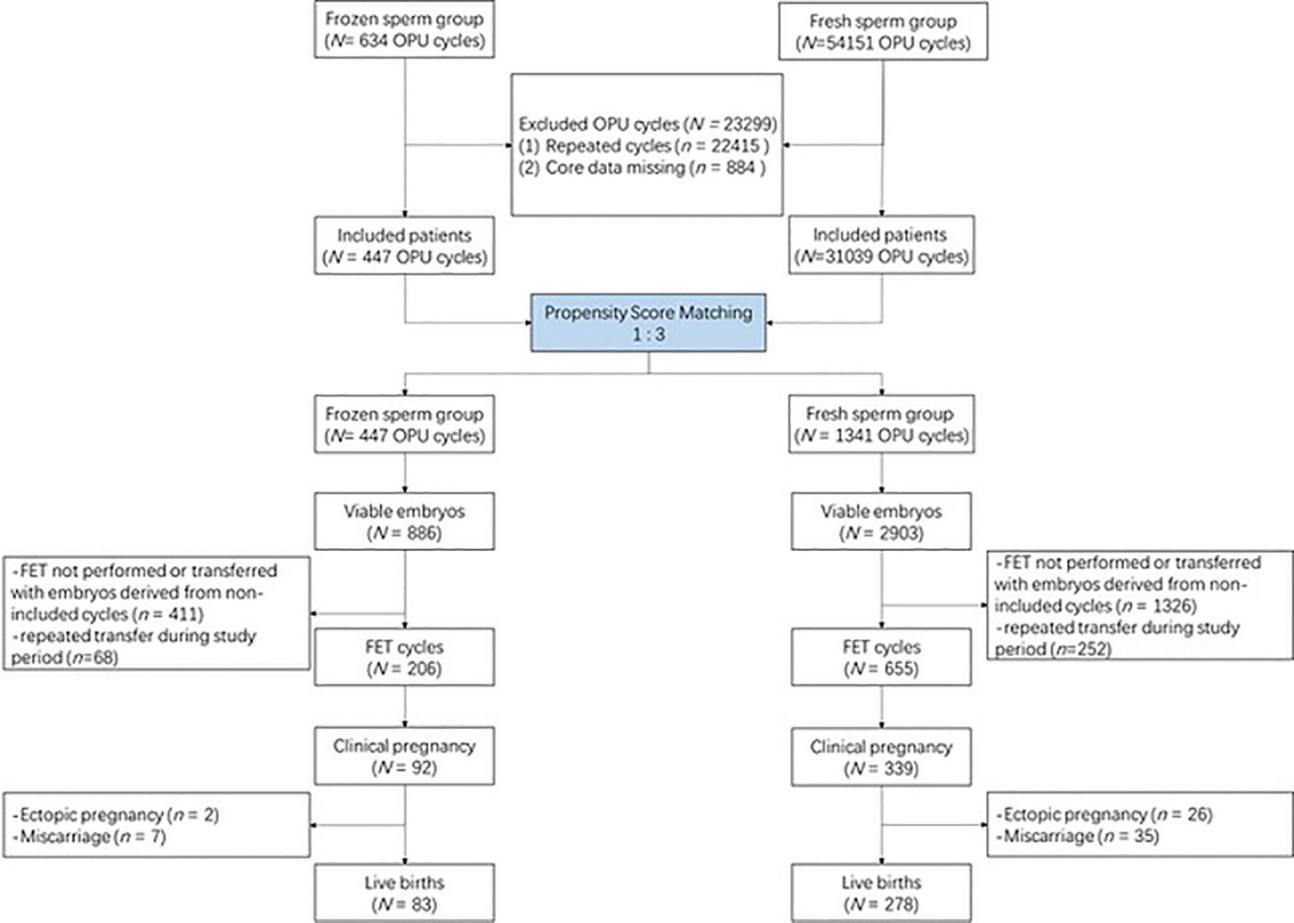
Flow chart of the study.

The flowchart of this study is presented in **Figure 1**. Briefly, a total of 54,785 cycles using IVF were screened from our database. After excluding 22,415 repeated cycles and 884 cycles whose core data were missing, 31,486 cycles remained. A total of 447 cycles with frozen sperm were matched with 1,341 cycles with fresh sperm at a ratio of 3. **Supplementary Figure S1** shows the distribution of propensity scores before and after matching.

### 3.1 Baseline characteristics

As demonstrated in **Table 1**, no significant between-group differences were found in the post-matching analysis with regard to all baseline characteristics, including female age, male age, female body mass index, infertility type, infertility duration, female infertility diagnosis, gravidity, parity, treatment protocol, OPU year, as well as sperm concentration and motility before preparation (all *P >*0.05).

**Table 1:** Baseline characteristics by group.

	Frozen sperm (*N*=447)	Fresh sperm (*N*=1,341)	*P*-value
Female age	36.85 ± 5.48	36.81 ± 5.50	0.908
Male age	39.35 ± 6.90	39.35 ± 6.71	0.778
Female body mass index	21.86 ± 3.22	21.78 ± 3.04	0.722
Primary infertility	172 (38.5)	524 (39.1)	0.867
Duration of infertility	4.21 ± 4.00	4.15 ± 3.97	0.811
Female infertility diagnosis			0.928
Tubal	126 (28.2)	369 (27.5)	
Mix/other factors	291 (65.1)	886 (66.1)	
Unexplained	30 (6.7)	86 (6.4)	
Gravidity ≥1 (%)	273 (61.1)	809 (60.3)	0.823
Parity ≥1 (%)	80 (17.9)	242 (18.0)	1.000
Treatment protocol (%)			0.966
PPOS	232 (51.9)	722 (53.8)	
Mild stimulation	93 (20.8)	276 (20.6)	
GnRH-ant protocol	31 (6.9)	81 (6.0)	
Long protocol	38 (8.5)	103 (7.7)	
Short protocol	26 (5.8)	78 (5.8)	
Natural cycle	27 (6.0)	81 (6.0)	
OPU year (%)			0.790
2011–2013	150 (33.6)	425 (31.7)	
2014–2015	149 (33.3)	443 (33.0)	
2016–2018	110 (24.6)	342 (25.5)	
2019–2021	38 (8.5)	131 (9.8)	

Data are presented as mean ± standard deviation or number (percentage).

PPOS, progestin-primed ovarian stimulation; OPU, oocyte pick-up.

### 3.2 Laboratory outcomes

In our center, sperm were frozen before the preparation step. After thawing, the sperm would be measured first, prepared, and measured again. As presented in **Table 2**, sperm motility decreased from 82.04% to 75.70% (*P* < 0.001) after the freezing–thawing process, while the sperm concentration decreased slightly from 30.92 to 29.68 × 10^6^/mL (*P* = 0.001). After *in vitro* fertilization, the fertilization rates between the two groups were comparable (68.27% for frozen sperm and 67.54% for fresh sperm, *P* = 0.704). The number of viable embryos also was not impaired by the freezing–thawing process of sperm (1.98 for frozen sperm and 2.16 for fresh sperm, *P* = 0.168). Similarly, there were no significant between-group differences when the number and rates of good embryos and blastocysts were analyzed (all *P* > 0.05).

**Table 2:** Cycle characteristics and outcomes by group.

	Frozen sperm (*N*=447)	Fresh sperm (*N*=1,341)	*P*-value
Sperm characteristics			
Concentration before preparation × 10^6^/mL	66.17 ± 22.93	66.87 ± 22.30	0.128
Motility before preparation (%)	31.61 ± 12.83	31.88 ± 10.72	0.166
Concentration after preparation × 10^6^/mL	29.68 ± 14.09	30.92 ± 12.77	**0.001**
Motility after preparation (%)	75.70 ± 19.42	82.04 ± 17.01	**<0.001**
Embryonic characteristics			
No. of oocytes retrieved			0.997
1–3	205 (45.9)	618 (46.1)	
3–10	182 (40.7)	541 (40.3)	
11–15	36 (8.1)	107 (8.0)	
≥16	24 (5.4)	75 (5.6)	
No. of normal fertilized oocytes	2.93 ± 3.41	3.21 ± 3.68	0.184
Normal fertilization rate (%)	68.27 (35.89)	67.54 (36.14)	0.704
No. of viable embryos	1.98 ± 2.38	2.16 ± 2.47	0.168
Viable embryo rate per oocyte retrieved (%)	44.26 (37.33)	44.76 (37.07)	0.721
No. of good embryos	1.70 ± 2.13	1.95 ± 2.56	0.104
Good embryo rate per oocyte retrieved (%)	38.77 (37.69)	39.65 (36.80)	0.481
No. of viable blastocysts	0.32 ± 0.85	0.33 ± 0.91	0.789
Blastocyst rate per embryo cultured for blastulation (%)	15.98 (26.87)	17.37 (30.47)	0.988

Data are presented as mean ± standard deviation or number (percentage). Sperm were prepared after freezing–thawing.

IVF, *in vitro* fertilization. P < 0.05 was considered as statistically significant and shown in bold.

### 3.3 Pregnancy outcomes

**Table 3** shows the pregnancy outcomes of frozen–thawed embryos derived from included OPU cycles. No between-group differences were observed in the number of transferred embryos, embryo stage at transfer, and endometrial preparation protocol. Among 206 cycles using frozen sperm, 102 cycles (49.5%) led to clinical pregnancy, while 369 cycles (56.3%) of 655 cycles using fresh sperm resulted in clinical pregnancy (*P* = 0.102). The biochemical pregnancy rate and implantation rate were also comparable between the frozen sperm and fresh sperm groups (all *P* > 0.05). Similarly, the live birth rate per FET cycle was 40.3% in the frozen sperm group and 42.4% in fresh sperm cycles (*P* = 0.642).

**Table 3:** Pregnancy outcomes of frozen–thawed embryos originating from the two regimens.

	Frozen sperm	Fresh sperm	*P*-value
No. of cycles	206	655	
Cycle characteristics			
No. of embryos transferred per cycle			0.876
Single	60 (29.1)	185 (28.2)	
Double	146 (70.9)	470 (71.8)	
Embryo stage at transfer			1.000
Cleavage stage	180 (87.4)	574 (87.6)	
Blastocyst stage	26 (12.6)	81 (12.4)	
Endometrial preparation			0.815
Mild stimulation	66 (32.0)	207 (31.6)	
Hormone replacement therapy	87 (42.2)	265 (40.5)	
Natural cycle	53 (25.7)	183 (27.9)	
Pregnancy outcomes per cycle			
Biochemical pregnancy rate	102/206 (49.5)	369/655 (56.3)	0.102
Clinical pregnancy rate	92/206 (44.7)	339/655 (51.8)	0.090
Implantation rate	121/352 (34.4)	422/1125 (37.5)	0.317
Miscarriage rate	7/92 (7.6)	35/339 (10.3)	0.561
Live birth rate	83/206 (40.3)	278/655 (42.4)	0.642

Data are presented as number (percentage).

FET, frozen–thawed embryo transfer.

### 3.4 Neonatal outcomes

As demonstrated in **Table 4**, 62 singletons and 42 twins were born from frozen sperm, and 219 singletons and 118 twins were born from fresh sperm. In both singletons and twins, no between-group differences were observed in offspring gender, gestational age, and birthweight (all *P* > 0.05). For adverse neonatal outcomes, the comparisons did not reveal any significant differences in preterm birth (<37 weeks), low birthweight (<2500 g), and major congenital malformation (all *P* > 0.05).

**Table 4:** Neonatal outcomes of live-born infants.

	Singletons			Twins		
	Frozen sperm	Fresh sperm	*P*-value	Frozen sperm	Fresh sperm	*P*-value
No. of children	62	219		42	118	
Male offspring	33 (53.2)	113 (51.6)	0.934	20/42 (47.6)	60/118 (50.8)	0.857
Gestational age (weeks)	38.13 ± 1.78	38.61 ± 1.41	0.126	36.00 ± 2.61	36.14 ± 1.78	0.954
Birthweight (g)	3,219.03 ± 511.23	3,312.83 ± 544.33	0.278	2,458.40 ± 582.22	2,520.08 ± 456.29	0.903
Adverse neonatal outcomes, *n* (%)						
Preterm birth (<37 weeks)	9 (14.5)	13 (5.9)	0.051	9/21 (42.9)	23/59 (39.0)	0.959
Low birthweight (<2,500 g)	3 (4.8)	13 (5.9)	1.000	17/42 (40.5)	41/118 (34.7)	0.634
Major congenital malformations	0 (0.0)	2 (0.9)	1.000	0/118 (0.0)	1/118 (0.8)	1.000

Data are presented as mean ± standard deviation or number (percentage).

## 4 Discussion

In this retrospective study, we found that although sperm motility was impaired after the freezing–thawing process, frozen–thawed sperm led to that are comparable with those of pregnancy and neonatal outcomes fresh sperm.

### 4.1 Strength and weakness

The sample size of 447 cycles using frozen–thawed sperm for IVF is the largest, to our best knowledge, in this topic. Besides that, propensity score matching, a highly specialized follow-up system, and careful selection of the study population made the statistical model more reliable and less biased.

However, this study is limited by the retrospective design, which cannot exclude unknown confounders. Prospective studies with a larger sample size are needed to confirm our results. Another limitation is that the results could be restricted to frozen–thawed embryo transfer cycles.

### 4.2 Comparison with previous studies

Techniques for sperm cryopreservation have been widely used in patients with azoospermia and severe oligozoospermia. Many studies have analyzed the effect of frozen sperm for ICSI in these patients, and three meta-analyses have summarized them well. The first one analyzed 17 researches with 1,476 ICSI cycles performed before 2004 in patients, with obstructive or non-obstructive azoospermia (17) and showed that, compared with the fresh sperm group, the clinical pregnancy rate decreased in the frozen–thawed epididymal sperm group (RR: 1.20; 95% CI: 1.0–1.42) and the implantation rate decreased within the frozen testicular sperm group (RR: 1.75; 95% CI: 1.10–2.80), while the fertilization rates were comparable among the three groups (17). The second meta-analysis including 574 ICSI cycles in patients with nonobstructive azoospermia was published in 2014 and reported no difference in any clinical outcomes between frozen and fresh sperm (15). The latest one including 17 studies with 1,261 cycles was performed in 2018 and also reported a similar result—that is, cryopreservation did not affect the fertilization rate or the live birth rate (16).

The above mentioned evidence has indicated that using frozen–thawed sperm in ICSI might not affect the outcomes in patients with azoospermia. However it still cannot represent the effect on IVF in patients with normal sperm. First, sperm from patients with obstructive or non-obstructive azoospermia was usually retrieved from the epididymis or testis instead of by masturbation. Thus, we cannot exclude the possibility that the difference in terms of sperm origin would cause different tolerance levels to cryopreservation. Besides that, IVF requires higher sperm quality, which might be seriously impaired during the freezing–thawing process.

Another major application of frozen–thawed sperm is fertility preservation in cancer patients, which helps patients freeze their sperm before antineoplastic therapy and receive ART treatment later. Due to the specific study population of cancer patients, the sample sizes were usually very small. One study with 30 ICSI cycles reported that half cycles led to clinical pregnancies finally, which is comparable to the control group only with tubal factor infertility (22). Another three studies demonstrated differences between IVF and ICSI. The first one including 29 patients demonstrated slightly higher fertilization rate and pregnancy rate in ICSI cycles than in IVF cycles and ultimately five live births from the 26 IVF cycles (19.2%) and four from the 19 ICSI cycles (21.1%) (19). The second study including 75 cycles reported a similar fertilization rate (49% vs 51%) but with a lower live birth rate (34% vs 40%) in IVF than in ICSI (12). The last one with a comparatively larger sample size observed that the fertilization rate and delivery rate of frozen sperm dropped by half in 54 IVF cycles than in 169 ICSI cycles, and they also reported a comparable pregnancy rate of 118 cancer patients (56.8%) using sperm frozen before therapy to other male-factor patients in ICSI cycles (11).

Contrary to previous studies, frozen–thawed sperm led to fertilization rates and live birth rates that are similar with those of the fresh sperm group in this current study. Sperm included in the abovementioned studies were frozen many years ago for fertility preservation. The long-term cryopreservation for fertility preservation and the underdeveloped freezing–thawing procedure many years ago might be attributed for the impaired fertilization rate and subsequent live birth rate. Besides that, freezing–thawing techniques have developed rapidly over the years. New methods including antioxidants, vitrification, freezing in seminal plasma, and so on attempted to minimize the damage to sperm potential (23–26).

### 4.3 Possible mechanism

In line with previous studies, sperm motility was impaired after freezing–thaw process. The difference in sperm motility between the fresh and the frozen–thawed sperm is 6.34%. It should be noted that we evaluated frozen–thawed sperm motility after the isolation and swim-up procedure, and therefore this parameter could more accurately reflect the fertilization situation.

Many studies have described the possible mechanisms of freezing damage to sperm. Intracellular and extracellular ice crystals forming during the freezing–thawing process would damage organelle structure and function (27, 28). A Rhodamine 123-based study found that mitochondrial activity decreased by nearly half after the freezing–thawing process (29). Besides that, oxidative stress during cryopreservation is another vital factor that damages sperm quality (6, 7, 30). Decreased motility after thawing showed a close association with increased oxidation–reduction potential, which is related to damaged axonemal structure and plasma membrane (30). Moreover, many studies have reported that cryopreservation of sperm led to increased DNA fragmentation (7, 10, 31), which is related to an increased risk of miscarriage and lower live birth rate (26, 32–36).

Although freezing damage is still unavoidable nowadays, much literature reported that normal sperm is more resistant to freezing damage than poor-quality sperm. Donelly and colleagues compared the tolerance level of ejaculated sperm to cryopreservation in patients with and without male factor infertility (37). The DNA integrity of frozen–thawed sperm was impaired seriously (decreased by 24–40%) in infertile patients but stayed intact in fertile patients (19). Zhang *et al*. analyzed the relationship between the sperm parameters before cryopreservation and the recovery rate of progressive motility and found that the sperm quality, including concentration, progressive motility, and morphology, before freezing exhibited a positive correlation with the recovery rate of progressive motility after thawing (38). Studies in rhesus monkeys (39) and goats (40) also observed similar phenomena.

Lastly, the ultimate aim of sperm cryopreservation is to deliver a healthy baby. What does it matter if sperm motility is reduced as long as it does not affect embryo development and live birth? Eastick and colleagues used time-lapse microscopy to observe the development of embryos derived from fresh or frozen sperm and found a difference in the morphokinetic parameters and embryo development between the two groups (4). In this study, we prepared the sperm by isolation and swim-up to select and enrich healthy sperm. The motility of frozen–thawed sperm was only 6.34% lower than that of fresh sperm, which is also sufficient for successful IVF, and the final live birth rate was comparable. In the fresh sperm group, 655 transfers led to 278 babies (42.4%), while 206 transfers led to 83 babies (40.3%) in the frozen–thawed sperm group. Also, no between-group difference was observed in neonatal outcomes.

It should be noted that the live birth rate (41.9%) in this current study is much lower than the previous report (50.74%). The possible reason could be the advanced female age in this study (36.8 years old) than in the previous study (31.8 years old). The preference for frozen–thawed sperm in older couples probably resulted from clinical inconvenience and affordable payments. Therefore, instead of directly comparing patients using frozen or fresh sperm, we performed propensity score matching to minimize the differences in baseline characteristics and make the results more solid. Although the treatment and laboratory procedure remained consistent during the whole study period, we cannot deny the technique improvements in 10 years. To correct the effect of time, we included treatment year as a covariate into the matching model, and the primary results remained stable.

## 5 Conclusion

Frozen–thawed sperm resulted in embryonic and pregnancy outcomes comparable with those of fresh sperm in IVF and subsequent frozen–thawed embryo transfer cycles.

## Data Availability

The raw data supporting the conclusions of this article will be made available by the authors without undue reservation.
