# Supplementary material for: Effect of freezing and thawing on ejaculated sperm and subsequent pregnancy and neonatal outcomes in IVF
Source: Front Endocrinol (Lausanne). 2024 Dec 16;15:1408662. doi: 10.3389/fendo.2024.1408662 (PMC11684094; doi:10.3389/fendo.2024.1408662)
Supplement: Supplementary file 1 [file DataSheet1.docx]

Supplementary Material

# Supplementary Figures

**Supplementary Figure 1.** Distributions of propensity scores before and after matching.

**
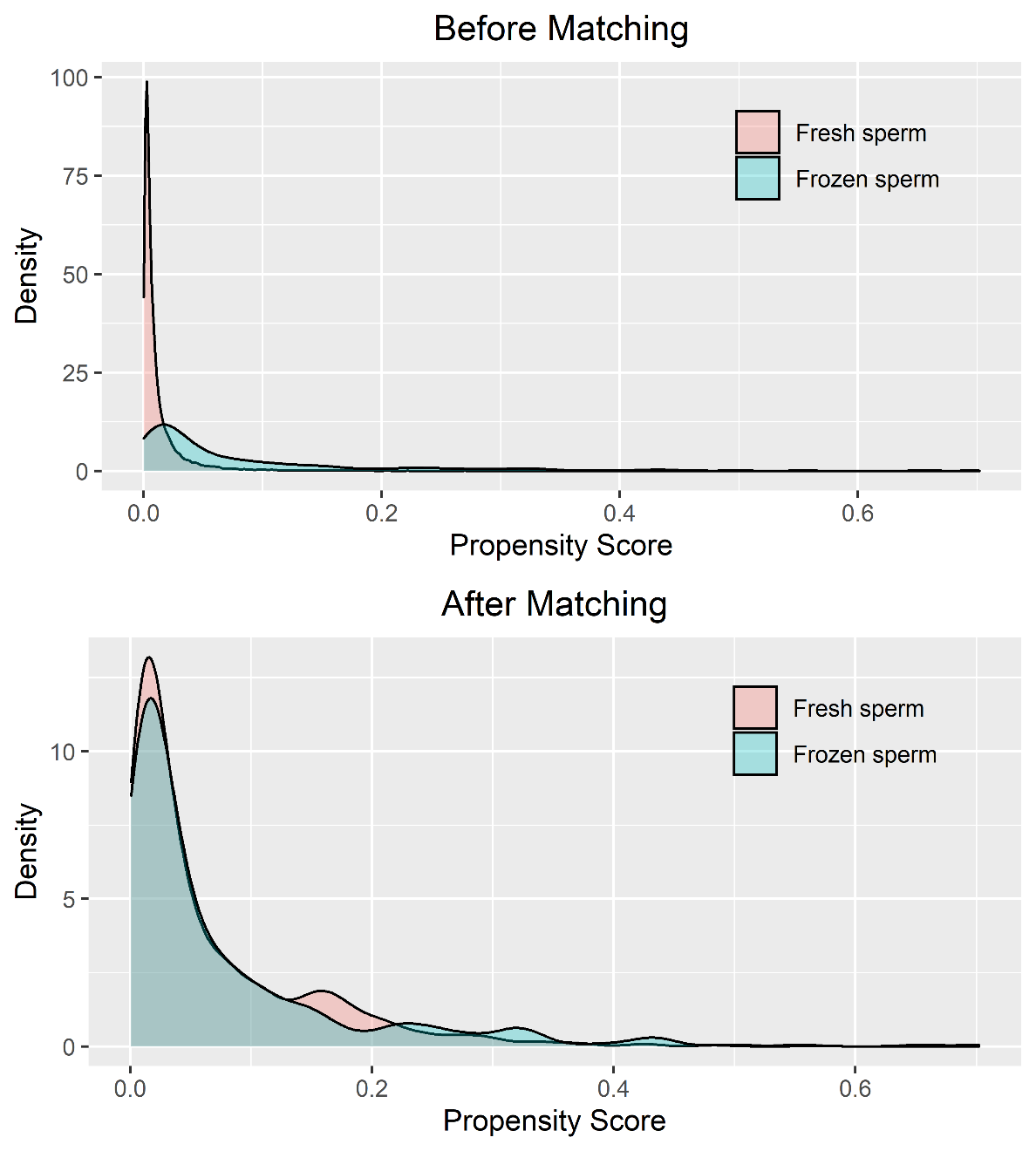
**
